# Supplementary material for: Prevalence and nature of self-reported visual complaints in people with Parkinson’s disease—Outcome of the Screening Visual Complaints questionnaire
Source: PLoS One. 2023 Apr 4;18(4):e0283122. doi: 10.1371/journal.pone.0283122 (PMC10072373; doi:10.1371/journal.pone.0283122)
Supplement: S1 Table — (PDF) [file pone.0283122.s001.pdf]

S1 Table. Ophthalmological conditions in people with PD and age-matched controls.

	People with PD (n = 581)		Control subjects (n = 583)	
	N	%	N	%
Cataract	151	26.0%	61	18.2%
Glaucoma	28	4.8%	0	0%
Macular degeneration	23	4.0%	0	0%
Strabismus/squint	17	2.9%	8	1.4%
Corneal abnormality (including pterygium)	13	2.2%	1	0.2%
Dry eyes disease	13	2.2%	1	0.2%
Macular abnormality	11	1.9%	2	0.3%
Ocular hypertension	9	1.6%	3	0.5%
Cloudy vitreous humor (including floaters/mouches volantes)	9	1.6%	1	0.2%
History of retinal detachment	7	1.2%	0	0%
(Recurrent) inflammation or uveitis	6	1.0%	0	0%
Retinopathy	5	0.9%	0	0%
Ocular migraine	5	0.9%	0	0%
Venous occlusion	5	0.9%	0	0%
Macular hole	4	0.7%	0	0%
Eye movement disorder	4	0.7%	0	0%
History of retinal defect	3	0.5%	2	0.3%
Sjögren's syndrome and keratoconjunctivitis sicca	2	0.3%	0	0%
Retinitis pigmentosa	2	0.3%	0	0%
Vitreous hemorrhage	2	0.3%	0	0%
Apraxia of eyelid opening	1	0.2%	0	0%
Nanophthalmia	1	0.2%	0	0%
Amaurosis fugax	1	0.2%	0	0%
History of melanoma in the eye	1	0.2%	0	0%
Ptosis	1	0.2%	0	0%
Graves' disease	0	0%	1	0.2%
Pinguecula	0	0%	1	0.2%

Note: this table is similar to the one describing the same population in a previous publication [13]. PD = Parkinson's disease
