# Supplementary material for: Prevalence and nature of self-reported visual complaints in people with Parkinson’s disease—Outcome of the Screening Visual Complaints questionnaire
Source: PLoS One. 2023 Apr 4;18(4):e0283122. doi: 10.1371/journal.pone.0283122 (PMC10072373; doi:10.1371/journal.pone.0283122)
Supplement: S3 Table — (PDF) [file pone.0283122.s003.pdf]

Screening Visual Complaints (SVC)

DISCLAIMER: The following questionnaire is a first English translation of the original Dutch questionnaire. This English version has not been validated yet and therefore does not guarantee similar validity and reliability as the Dutch version.

Date: ____

Name: ____

Sex: ____

Date of birth: ____

What is your highest level of education? ______________________________

The following list of questions concerns problems that you may have with your eyesight. If you wear glasses or contact lenses, please assume that you are wearing these when you answer the questions.
Each question has several possible answers. Please choose the answer that is most appropriate to your situation as it has been over the past weeks.
If you are not certain, please choose the answer that best reflects your situation.
Please choose 1 answer for each of the following questions. The questionnaire consists of 3 pages.

		Yes	No
	Did you ever visit an ophthalmologist?	❒	❒
	If 'Yes':<br>Which ophthalmologist did you visit or at which hospital did you visit an ophthalmologist?		
	For which ophthalmologic condition did you visit an ophthalmologist?		

		No/ Hardly ever	Sometimes	Often/ Always
1	Do you experience problems with your eyesight in daily life?	❒	❒	❒
	If 'Sometimes' or 'Often/Always': please describe your problems or complaints regarding your eyesight			
	a.			
	b.			

	c.			
	d.			
		**No/ Hardly ever**	**Sometimes**	**Often/ Always**
2	Do you have the impression that your vision has become less clear?	❒	❒	❒
3	Do you have trouble focusing or does it take longer before things are in focus?	❒	❒	❒
4	Do you have double vision or see double images?	❒	❒	❒
5	Do you have problems with depth perception or estimating distances?	❒	❒	❒
6	Do you see shaky, jerky or shifting images?	❒	❒	❒
7	Do you have the impression that you cannot see part(s) of the visual field?	❒	❒	❒
8	Do you experience colour differently than before?	❒	❒	❒
9	Do you have trouble seeing things at reduced contrast (e.g. letters that have not been printed on a white but on a grey background)?	❒	❒	❒
10	Are you more easily blinded by bright light than before?	❒	❒	❒
11	Do you have the impression that everything looks darker or that you need more light than before?	❒	❒	❒
12	Do you have difficulty adjusting to light or dark environments?	❒	❒	❒

13	Do you see things that others do not see (e.g. flashes of light, patterns, objects or animals)?	❒	❒	❒
14	Do you have the impression that you perceive objects or faces differently, for example, distorted or with afterimages?	❒	❒	❒
15	Are your eyes painful?	❒	❒	❒
16	Are you bothered by dry eyes?	❒	❒	❒
17	Do you feel that you need more time to see things?	❒	❒	❒
18	Do you have vision problems when you participate in traffic (walking, cycling or driving)?	❒	❒	❒
19	Do you have trouble looking for objects and finding objects due to your eyesight?	❒	❒	❒
20	Do you have trouble reading due to your eyesight?	❒	❒	❒

		**Please indicate your answer on a scale of 0 to 10**<br>*(please circle the relevant answer)*
21	To what extent do you experience limitations in daily life due to the above mentioned problems with eyesight?<br><br>*0 = no limitations*<br>*10 = very severe limitations*	0 1 2 3 4 5 6 7 8 9 10

	Yes	No
Would you appreciate advice, assessment and/or rehabilitation for the abovementioned complaints?	❒	❒

Please check whether you answered all questions.
One answer must be ticked for each question.

Thank you very much. This is the end of the questionnaire.

Huizinga F, Heutink J, de Haan GA, van der Lijn I, van der Feen FE, Vrijling ACL, Melis-Dankers BJM, de Vries SM, Tucha O, Koerts J. The development of the Screening of Visual Complaints questionnaire for patients with neurodegenerative disorders: evaluation of psychometric features in a community sample. PloS One. 2020
