# Supplementary material for: Prevalence and nature of self-reported visual complaints in people with Parkinson’s disease—Outcome of the Screening Visual Complaints questionnaire
Source: PLoS One. 2023 Apr 4;18(4):e0283122. doi: 10.1371/journal.pone.0283122 (PMC10072373; doi:10.1371/journal.pone.0283122)
Supplement: Attachment — Submitted filename: Response to Reviewers.docx [file pone.0283122.s004.docx]

| **Comments of reviewers** | **Response of authors**  *(lines mentioned refer to the document including track-changes)* |
| --- | --- |
| Reviewer #1: | |
| It was interesting to learn from the Authors that PD patients are at high risk of visual complaints such as trouble to reading and focusing. These alterations are likely associated, at least partially, with oculomotor impairments since other visual attributes that may be less dependent on precise fixational eye movements, such as visual field and color vision, showed much lower prevalence.  […] I recommend to include in the discussion a brief description concerning the above-mentioned possible associations between visual disturbances and oculomotor alterations in PD patients that may be a subject of future investigations. | We added a paragraph on the possible relationship between the most common complaints and oculomotor deficits in the discussion section (lines 315-323). Furthermore, we stated that it might be interesting for future research to look into the possible relationship between visual complaints and functional disorders (lines 371-373). |
| Reviewer #2: | |
| In the introduction the authors can write more about the visual defects that are reported among PD patients with relevance to their stage of PD | We added a paragraph discussing what was known about visual complaints from the scientific literature. We also briefly discussed the relationship to certain disease-related data, such as disease severity (lines 54-62). |
| Whether any subsequent ophthalmic examinations were carried out on the self-reported patients to confirm the nature of the visual condition, like assessing their visual acuity to further confirm ? unclear vision appears to be a pre-dominant complaint with 23.2% reporting, so having a simple visual acuity test on these patients would have added more value to the data. | We did not include assessments of visual functions. We agree that possible underlying visual function deficits might have been interesting to look into. We have recommended this for future research (lines 371-373).  The rational for focusing on complaints in the current study was supported by the following: in some cases, the target of care could be an underlying functional impairment, as for example visual acuity (suggested by the reviewer), which might be addressed by the right glasses. In others, however, underlying functional impairments, as for example oculomotor deficits, cannot be treated well, while a complaint might still be addressed in rehabilitation (e.g., by advising a certain reading distance). There may not be a one-to-one association between objectified visual impairments and subjective visual complaints. We added a few sentences on this topic in the discussion section (lines 340-348). Since the reduction of complaints is the goal of rehabilitation, and knowledge on this topic is still scarce, we chose complaints as the subject of this study. |
| Did the authors consider analyzing the data from PD cases based on their stage of the disease apart from classifying based on their age, if so how was the correlation between the visual condition and stages ? This would provide some insight on the ophthalmic deterioration with relevance to the severity of the PD. | We did take disease severity into account, by calculating differences in the presence of visual complaints between people in several disease stages, according to Hoehn & Yahr (1998; see Table 6a and 6b). These analyses indeed show that visual complaints seem to increase with disease severity. We did not perform correlations. |
| How the co-morbidity of diabetes among the PD cases were considered ? and does the control group contains diabetes cases ? | Supplementary Table 1 presents the comorbid ophthalmological conditions in both groups. We reported diabetes in case there was proven retinopathy (n = 5 in PD, n = 0 in controls). In addition, this study investigated the relationship between the presence of visual complaints and the presence of ophthalmological conditions, including retinopathy. |
| The authors need to clarify how many of the PD cases reported here were under dopaminergic medication and also of the total PD cases reported how many were freshly identified to have PD and without taking any dopaminergic medication ? | We added four rows in Table 1, describing how many people with PD were taking the most common forms of antiparkinsonian medication and how many were not taking any medication. |
| Also it would be nice to record the sleep patterns of the PD patients especially those undergoing dopaminergic treatment and correlate with the visual condition ? | We agree that this might be interesting for future research. However, we feel this is beyond the subject of the present study. |
| Reviewer #3: | |
| Abstract: Please indicate the numerical prevalence in the results. | We included the exact prevalence of complaints in the abstract. |
| Introduction: The Introduction is short, I suggest a paragraph fastly citing the more important or known visual complaints. It would be informative for a general audience. | We added a paragraph discussing what was known about visual complaints from the scientific literature (lines 54-62). |
| Methods: Was there some information about the socioeconomic profile from PD patients and controls? If not, include as limitation. | We did not have access to information regarding the socioeconomic status of the participants. We included a statement on this in the discussion section (lines 413-416). |
| Results: Figure 1 has no legend in the Y- and X- axis, and the numbers have low contrast. I suggest to include the legends and paint in black the numbers and letters. Why is not indicated the statistical difference? | We adjusted the figure based on these comments. |
| Discussion: The methods of data collection in PD patients and controls were different and it is an limitation of the study. The authors should discuss about it. | We added a paragraph on this limitation in the discussion section (lines 392-398). |
|  |  |
| Reviewer #4: | |
| The title can be “Prevalence and …….. - Screening Visual Complaints questionnaire outcome” or "outcome of SV...Q"; instead of “Use of the Screening Visual Complaints questionnaire”. | We have changed the title based on the suggestion given. |
| Line 69, please define mild, severe, and moderate condition staging. | We referred to notes e-f below Table 1 for a definition (line 88). If the editor wants us to provide a complete list of conditions excluded in the control group, we are able to provide this list. |
| Your control group had mild PD (line 67)? How is that not affecting your comparison with those having PD? | The control group had no PD; we made this more specific in the manuscript (lines 85-86). |
| “Dutch-speaking people were invited” implies they can be of any ethnicity speaking dutch. Is ethnicity a factor affecting PD? (DOI: 10.3233/JPD-191763) | It is true that people in either group could be of any ethnicity, as long as they speak Dutch. We did not collect data on ethnicity, so we cannot draw conclusion regarding its relationship with visual complaints in PD. We included a statement on this in the discussion section (lines 413-416). |
| Are your study participants “people” or are they “patients” of the center? | The people with PD were seen for their disease (i.e., as “patients”) at the Parkinson Expertise Center. In accordance with APA 7^th^ (https://apastyle.apa.org/style-grammar-guidelines/bias-free-language/disability), we do not define these people solely by their disease, which is why we use the term “people with PD”. |
| Line 154, mentioning Evdk as a research assistant is unnecessary. “Two researchers” is enough. | We removed initials from this sentence (lines 176-177). |
| The authors themselves published a systematic review of “Self-reported visual complaints in PD” in 2022 (DOI: 10.3233/JPD-202324). Moreover, the svc questionnaire you have used is an existing one (with a similar sample size and control group DOI: 10.1371/journal.pone.0272559), used previously and the results are in the systematic review itself. Please explain in detail the need for this study and what additional this study is achieving, that others could not before using the same questionnaire. Basically, how did you overcome the limitations of previous studies (if any). | The results of the study using the SVCq (2022) are not included in the systematic review mentioned, as the review included studies that were published up to February 5, 2021. Furthermore, the previous study using the SVCq had a different purpose, which was to validate the questionnaire and examine the factor structure of the questionnaire in a sample of people with PD, rather than to investigate the prevalence and nature of visual complaints in this sample, as is the case in the present article.  The main advantages of the current study is that it investigated a wide variety of function and activity related visual complaints in a large cohort of people with PD, and compared their results to an age-matched control group. As was found by the review mentioned, only few studies have previously aimed for mapping the prevalence of a number of visual complaints in people with PD. Previous studies have mainly used small sample sizes to draw conclusions on only one or a few complaints, sometimes even without comparing it to controls. These are important limitations that are overcome in this study. |
| Line 84-85, “In addition, a clear and clinically relevant factor structure has been demonstrated in people with PD” is unclear, please restructure. | We have added a sentence to clarify the meaning of this factor structure (lines 103-105). |
| Although PD patients were previously diagnosed and taken from medical files, please explain the diagnostic criteria of PD and staging and also for the control group. | Diagnosis of PD was done based on the UK Parkinson’s Disease Society Brain Bank Diagnostic Criteria. We added this in the manuscript (lines 84-85). Staging of the disease was reported in Table 1, by means of the commonly used staging by Hoehn & Yahr (1998). The control group consisted of a community sample without PD. Therefore, reporting of diagnostic criteria or disease stage is not applicable. |
| Please explain in detail the ophthalmological examination conducted on the PD and control group. | We did not perform any ophthalmological assessments. Data on any present ophthalmological comorbidities were recorded from medical files in those with PD, and in those without PD (controls) this was based on self-report (see Supplementary Table 1). This procedure was explained in the manuscript (subheading ‘Determining the presence of ophthalmological conditions’, lines 148-155). |
| Including people both with and without cataract surgery in the PD and control group, are you introducing any kind of bias or not? (Although you have mentioned excluding them did not alter the results). Please discuss this. | We explained this more clearly in the manuscript (lines 154-155). We cannot conclude that cataract surgery relieved all complaints, given the possibility of residual long-term effects or secondary cataracts. Therefore, we considered those who underwent surgery to have an OC. Since there was no difference in results after excluding people who had undergone cataract extraction, we do not expect bias of any kind. |
| Conclusion: “Since visual complaints can have a vast impact on the daily lives of people with PD” Have you tested the QOL in people with PD in this study? Either add a reference or remove the line. | This quote refers to the impact of visual complaints on the daily lives of people with PD, which we investigated through the present study (impact on daily life score, 0 - 10 scale). There is no mention of QOL in the cited quote from the conclusion. |
| Tables 7 and 8 look unnecessary. Adding results is enough. | Since reviewer #5 argues these tables contain information not previously studied and reported, we left these tables included in the manuscript. |
| Reviewer #5: | |
| First, I think both the introduction and discussion need some work. It is clear that this is an underrecognized topic and in need of more study. However, I think that these sections lack a good representation of the work that has already been performed on this subject. The introduction should, in addition, also indicate what this study adds to the current literature. | We added a paragraph in the introduction discussing what was known about visual complaints from the scientific literature and what the current study adds to this (lines 54-62 and 70-80). In the discussion section, we also made more links between our results and previous studies (e.g., lines 299-304 and 315-323). |
| What is truly new (in my opinion tables 5-8) and how could this help to improve health care? Do we need screening by the ophthalmologist based on this study? And on which ophthalmological diseases? It is difficult to treat only complaints without knowing what the underlying disorders might be. | In particular, screening for a wide range of function and activity related visual complaints in a large group of people with PD is something that has not been done. The use of the SVCq allows for screening for isolated complaints focused on both functions and activities, which provides clear goals for care and rehabilitation. We made the added value of this study more clear (e.g., lines 70-80).  As this study also shows, visual complaints are not fully explained by ophthalmological conditions. Complaints may also be present in their absence. In case complaints are present, screening for ophthalmological conditions or functional deficits can be done. However, even in the absence of these deficits, visual rehabilitation can be applied to relieve complaints (e.g., by providing aids or advice for activities that a person has difficulty with). We explained this in more detail in the discussion section (lines 340-348). In general, the current study advocates that regular screening for visual complaints is needed so that they are recognized and people can receive the right advice and rehabilitation, regardless of the cause. |
| It is stated in the discussion that some complaints (most seen in PD) are possible PD pathology related. This is interesting however there is no explanation on this topic. | There is a short explanation on this in lines 327-330. We state that for example PD related disturbances in retinal, cortical or thalamic functioning might contribute to the experience of visual complaints (supported by DOI: 10.1093/brain/aww175). In addition, we showed that complaints increase with disease progression. |
| In the discussion section, also a more in-depth discussion of the causes and consequences of the findings and a comparison with previous studies should be made. Now only the rationale that screening is important because of a high prevalence and a potential impact on daily life, is repeated several times in both the introduction and discussion. | We complemented the discussion with more links between our results and previous studies. In addition, we focused more on possible causes and consequences of our findings (e.g., lines 299-304, 315-323, and 340-348). |
| The SVCq is introduced as a questionnaire that screens for visual complaints in people with neurodegenerative diseases. However, the questionnaire was validated in people without neurological conditions. Please explain. | Even though developed to screen for visual complaints in people with neurodegenerative disorders, the items in the SVCq are general formulations of visual complaints that anyone can experience, not just people with a neurodegenerative disorder. As a first step, the SVCq was examined in a group of people without these disorders. Subsequently, we tested the found structure of the questionnaire in people with PD. Here we found similar results and clinical merit for use of the SVCq in people with PD (DOI: 10.1371/journal.pone.0272559). This is also explained in lines 102-105. |
| How was the question on dry eyes established? Dry eyes syndrome is a diagnosis and not typically a visual complain. Dry eyes could lead to symptoms of paradoxically to watery/teary eyes, burning/sandy/itchy eyes and/or blurred vision. | All items in the questionnaire were established based on literature (in which “dry eyes” were also regularly questioned; DOI: 10.3233/JPD-202324), expert opinions (e.g., neurologists and ophthalmologists working with neurodegenerative disorders, including PD), and experiences of people with a neurodegenerative disorder, including PD. A detailed description of the questionnaire development can be viewed at DOI: 10.1371/journal.pone.0232232. |
| The conclusion drawn in the discussion that the SVCq is very different from other questionnaires such as the VFQ-25 and the VIPD-questionnaire is not clear to me. When compared especially the VIPD-Q and the SVCq many questions are similar, for example distorted images, hallucinations, painful eye, contrast, color vision, double vision. Also, the frequency never, often etc. is very similar. Could the authors explain what the added value of the SVCq is? I agree that structured questioning of visual complaints should become routine care. | We tried to clarify this more in the discussion (lines 362-373). Despite several complaints appearing in both questionnaires, the VIPD-Q aims to detect underlying ophthalmological conditions. Questions are therefore for example phrased more specifically (e.g., does a complaint occur in a specific situation?). The SVCq asks about isolated complaints, at both a functional and activity level. As we found that complaints do not always directly relate to ophthalmological conditions, the aim of the SVCq is not to detect these conditions, but to guide care and rehabilitation. |
| It seems like the severity of complaints score (on a scale of 1-10) was not used in the analyses. The indications ‘never/ hardly’etc. were used to classify severity. Please explain. | We used this score as the ‘impact on daily life score’. Throughout the manuscript, we indicated more clearly where this score was used, by mentioning the 0 - 10 scale. |
| A limitation of the present study is the lack of objective data on ophthalmological disorders. Even though medical files were studied, one of the problems is that ophthalmological disorders are not recognized (and diagnosed) in PD in clinical practice (also see a recent publication of Borm et al. J Neurol 2022). So, the number of OC’s may have been underestimated. In addition, there may still have been some selection bias because only PD patients visiting the outpatient clinic of a university hospital were included. This is a selected population. | We agree that these are possible limitations of the study, which we addressed in the discussion section (lines 387-391 and 399-403). |
| The authors state following: “Therefore, there is a risk of underestimation in both groups. The percentage of people who indicated having visited an ophthalmologist did not differ between the groups (see Table 1). It is therefore unlikely that results were influenced by missing a large number of ophthalmic conditions in one of the groups.” This statement confused me, since underreporting of complaints is a reason to screen for visual complaints in PD, especially regarding the results of this study with high prevalence of visual complaints in persons with PD. Then it is paradoxal to assume that persons with PD did visit the ophthalmologist with their complaints earlier. Shouldn’t persons with PD show a much higher rate of visiting an ophthalmologist? Could the authors explain this? | Thank you for pointing this out. It is indeed incorrect what was stated here. We chose to omit the last two sentences, but to leave the limitation about the possible underestimation of the number of ophthalmic conditions (lines 399-405). |
| I would have liked to see analyses on the different age categories. A point of discussion is whether the high number of visual complaints and disorders in PD is a consequence of the disease or related to ‘general ageing’. It would be interesting to distinguish the age categories that were matched and perform the prevalence analyses for these different age categories. | In this study, we determined a positive correlation between the presence of visual complaints and age. We have also shown that people with PD experienced more visual complaints than controls. Given that these controls were age-matched to the group of people with PD, it can be concluded that the high prevalence of visual complaints in people with PD cannot be fully explained by ageing. We do agree that analyzing each age category may provide even more insight into possible interaction effects. As we already performed many analyses in this study, all of which include important variables, we do not feel that adding another set of analyses on just one of those variables is justified. Nonetheless, if the editor wants us to add these analyses, we can do that. |
| Were co-morbidities in the PD group considered in the analyses in a way? | We did not correct for comorbidities other than ophthalmological conditions. For this reason, there is a paragraph on the possible effect of this decision in the discussion section (lines 406-412). |
| In the discussion it is stated that the high prevalence of visual complaints in people with PD may negatively impact daily life. Wasn’t this also studied here? In general, I find the analyses and conclusions on the clinical impact difficult to interpret; this could be described more clearly and explicitly. | This was studied indeed, by the ‘impact on daily life score’ (0-10). Conclusions regarding impact on daily life were based on this score. We indicated more clearly where this score was used throughout the manuscript, facilitating interpretation. |
