# Supplementary material for: Prevalence and nature of self-reported visual complaints in people with Parkinson’s disease—Outcome of the Screening Visual Complaints questionnaire
Source: PLoS One. 2023 Apr 4;18(4):e0283122. doi: 10.1371/journal.pone.0283122 (PMC10072373; doi:10.1371/journal.pone.0283122)
Supplement: Attachment — Submitted filename: Response to reviewers.docx [file pone.0283122.s005.docx]

| **Comments of reviewer/editor** | **Response of authors**  *(lines mentioned refer to the document including track-changes)* |
| --- | --- |
| Reviewer #1:  The discussion regarding the effects of abnormal eye movements on visual functions in PD has been added by the Authors. Thank you! | We are pleased that our addition is sufficient. Thank you for the feedback. |
| Reviewer #3:  Dear Editor, all my issues were addressed and I considered that the manuscript is suitable to be published | Thank you for the compliment. |
| Reviewer #4: (No Response) | - |
| Reviewer #5:  I would like to thank the authors for addressing all comments of 5 reviewers. Most of my addressed points have been clarified. Although, I still have concerns about the final conclusion that there are complaints without underlying OC. In my opinion it is not possible to state this as part of the end conclusion without thorough ophthalmological assessment. for example impaired color vison could be related to retinal dysfunction this is an OC (due to parkinson's or not) the same for dry eyes, blurred vision due to refractive errors and reading problems due to oculomotor problems or visual field deficitis. Of course sometimes you don't know what the underlying cause to symptoms is and one can speculate it is parkinson's, but to state this with only self-reported OCs is difficult to accept for me. Especially noticing that PD patients underreport symptoms and therefore not go to eye care as often as they should. | We removed the conclusion on ophthalmological conditions from the abstract and conclusion, as this was not the main aim of our study, and we agree that these conditions were not extensively examined in our study as we used retrospective medical file investigation and self-report (lines 23-39, 425). Since we removed these results from the abstract, we elaborated a bit more on results related to our research questions on the prevalence and nature of visual complaints in people with Parkinson’s disease.  Furthermore, we chose to discuss the possible underestimation of ophthalmological conditions earlier in the discussion section, to enable readers to interpret the results in view of this information. We also emphasized that conclusions regarding analyses with ophthalmological conditions should be made with caution (lines 313-318).  In addition, we replaced ‘ophthalmological conditions’ with ‘identified ophthalmological conditions’ in the discussion section to indicate that we are referring only to what is known to us from medical files and self-reported information. With this, we make it even more clear that we cannot state with certainty that no other conditions were present (lines 310-312, 378). |
| Editor:  Thank you for submitting your valuable work.  The reviews, which are insightful and interesting, pointed to some unexplained aspects and the authors were able to clarify them.  By my own reading, the manuscript still needs a bit of refinement, mostly related to generalisation, conciseness and the control of confounding factors. Although this may sound counterintuitive, I am keen to understand authors' claims and keen on reading a refined manuscript.  Briefly, as raised by one of the reviewers, you cannot just make too much speculation of your findings.  As we have observed through this time, PD's ocular manifestations are measured by the use of behavioural and physiological techniques. For example, we have noted that the dopamine depletion can affect eye's structure but also functional aspects from the retina to brain (based on ERPs, colour or chromatic discrimination etc.) This is not the focus of the manuscript, indeed, but those findings showed that other measures are reliable enough to detect ocular and visual manifestations, but also help, somehow, during the course of the disease. My concern here is: you are using a self-reported measure to characterise visual complains and, hence, because of that, you cannot just speculate that much with strong statements or even with some assertions. I think the authors can work on a little bit, refining the manuscript to ensure that readers and researchers can easily understand that your claims and assertions are tightened up to your results - without too much "physiology" or "all or nothing" claims.  You will notice that reviewers provided frank and clear comments and, in this round, they almost had no concerns. This is a good point, actually, and the authors can rest assure that this will be considered. However, more important is to provide a frank, clear and transparent perspective of your findings. I'd highly recommend the authors to rethink some statements where sound like "causal" relation of the self-related and visual complains - that should be interpreted as self-reported.  For example, one could say that he/she has no visual complain. But when comes to the physiological aspect, them could have cortical or subcortical damages. Well, by self-report them reported nothing, but physiologically, them have.  So basically I am gentle asking you to be cautious in this aspect and also refine your limitations pointing this difference of self-reported and other measures that really can show causal relation of the complains. Your study has merits and should be considered, but, considering these aspects, please opt to use a more conservative approach for debating your data and for your conclusions.  Overall, the manuscript reads better and has been really refined considering all of the reviewers’ comment. Also I commend the authors for their politeness and efforts. Wishing you success with the study and a very good refined version, so then we can proceed. | Thank you for your compliments and feedback. We agree that in a previous version we drew some conclusions that were too strongly formulated. We may have speculated too much, and it was not always clear which results were found in our study and which in previous studies. Also, we may have unintentionally suggested some causal relationships. To correct this, we changed the following (in addition to what was mentioned above in the response to reviewer #5):   - We nuanced statements on complaints being present without ophthalmological conditions, by emphasizing that it is possible that complaints are related to ophthalmological conditions, as this is also suggested by results of our study. As our results raise the possibility that complaints may be present when there is no identified ophthalmological condition, we emphasize that screening for visual complaints is important in conjunction to screening for ophthalmological conditions or functional disorders (lines 375-379). - We replaced the words "explained" or “contributed to” with "related to" or “associated with”, to indicate that we did not demonstrate any causal relationships with our study (lines 309-337). - We made it more clear that we did not investigate any other factors besides ophthalmological conditions that may relate to experiencing visual complaints. We raised this as a possible topic for follow-up studies (lines 310-339). - We described more clearly which results emerged from our study and which from previous studies (lines 310-339). - We nuanced statements and conclusions and very tentatively suggest what our study can contribute to the management of visual problems in clinical practice (lines 346-355). |
