# Supplementary material for: Prevalence and nature of self-reported visual complaints in people with Parkinson’s disease—Outcome of the Screening Visual Complaints questionnaire
Source: PLoS One. 2023 Apr 4;18(4):e0283122. doi: 10.1371/journal.pone.0283122 (PMC10072373; doi:10.1371/journal.pone.0283122)
Supplement: Attachment — Submitted filename: Response to reviewers.docx [file pone.0283122.s006.docx]

| **Comment of editor** | **Response of authors**  *(lines mentioned refer to the document including track-changes)* |
| --- | --- |
| Editor:  As mentioned before, these (10.1371/journal.pone.0272559 & https://doi.org/10.1371/journal.pone.0232232) are studies that could be considered as potential overlapping. I ask the authors to detail in the main text the main differences - with specific details re. the three studies. Also, I request the authors to disclose in a diffrent section of the file, next to the diclosures and/or conflict. | We agree that it may not have been clear throughout the manuscript that similar datasets have been used previously to answer different research questions. For transparency reasons, we have revised our manuscript, and we hope the revisions clarify that previous studies focused on validating the questionnaire, whereas the current study aims to examine the prevalence of visual complaints in people with Parkinson's disease versus controls, based on the validated questionnaire, but using data from the same samples.  We adjusted the following:   - We cited both previous studies in the introduction, describing what the goals of those studies were as well as the goals of the current study, which do not overlap (lines 72-79). - We are transparent about including the same samples in the current study and added a reference to previous studies on any occasion we discuss the samples. However, if you think we are now overdoing the number of references (self-citation), please let us know. - We added a disclosure to the manuscript stating that data from the samples was published previously.   We would also like to mention that results of the previous studies were summarized in the method section (subheading ‘Screening Visual Complaints questionnaire’; lines 103-108) and results presented in this study have not been published elsewhere. |
