# Supplementary material for: Prevalence and nature of self-reported visual complaints in people with Parkinson’s disease—Outcome of the Screening Visual Complaints questionnaire
Source: PLoS One. 2023 Apr 4;18(4):e0283122. doi: 10.1371/journal.pone.0283122 (PMC10072373; doi:10.1371/journal.pone.0283122)
Supplement: Attachment — Submitted filename: Response to Reviewers.docx [file pone.0283122.s007.docx]

Dear Dr. Thiago Fernandes,

Enclosed you will find our response to your comments. The most important point we address here is that this paper and the previously published paper on the factor structure of the Screening Visual Complaints questionnaire (SVCq) in PLOS ONE have completely different and equally relevant aims, and perform different analyses, using the same dataset.

As indicated at the time of initial submission, data used in the current manuscript were previously used for a publication on the construct validity of the SVCq (see doi.org/10.1371/journal.pone.0272559). The current paper builds on the previous one and has used the SVCq to analyse the prevalence of visual complaints in a well-defined PD cohort. Hence, we consider our previous publication as an essential first step, before we could conduct the current analyses on the prevalence of visual complaints. We followed a similar path with our research in people with multiple sclerosis (MS). The first paper was also on the construct validity of the SVCq in people with MS, whereas the second paper published the actual prevalence of visual complaints in people with MS using this SVCq (see doi.org/10.1186/s41687-022-00443-0 and doi.org/10.1016/j.msard.2021.103429). We would like to stress that that dividing these topics over two publications should not be considered “salami slicing”. The tables in the current publication report prevalence figures, whereas our previous publication is discussing which structure of the SVCq would provide the most differentiated and solid outcomes of this scale.

Since part of your concerns refer to the re-use of data, we would like to address that according to the international guidelines for scientific journals, editors and researchers, the use of previously published data for a new publication is accepted under the following circumstances:
- it should be clear that the dataset has been previously published and reference is made to that previous publication through citations.
- the same dataset is used to answer different research questions.
- research questions are answered with a different statistical method.
- the same dependent and independent variables are used, both in number and in denomination.
- the results and conclusions clearly complement the previous publication.

Following the above-mentioned international guidelines, we now have clearly indicated that we used the same dataset as our previous PLOS ONE publication, but different parts (subscores) of it, to answer different and complementing research questions (see our Introduction lines 76-83 and Methods section lines 87-88; changes mentioned in this letter refer to the document including track-changes) and results (see Discussion lines 365-371). The main components of the studies are essentially different: the previous study performed a factor analysis on data from people with Parkinson's disease, whereas the current study calculates frequencies of visual complaints in people with Parkinson's disease, compared to controls. We now added in the Method section that analyses and results of the five subscales can be found in our previous PloS One publication (line 199), clarifying what new information the current paper adds to the previous. More importantly, the prevalence of individual visual complaints covered by the SVCq has not been previously published, thus making all results in the current manuscript new.

Although we appreciate your suggestion to add new data to the current dataset, we do not think this is a good idea. First, adding new data to an existing dataset that has been used for a previous publication could be regarded as augmenting a dataset to mask overlap. The new (augmented) dataset and the previously used dataset would still have the same amount of overlap, albeit a slightly smaller proportion of overlap. Second, the risk that readers and researchers might consider the two datasets to be different is present, and consequently both datasets might be included in a meta-analysis, which would be wrong. A more practical reason is that obtaining new ethical approval, approaching and selecting new participants with Parkinson’s disease from the same population in the North of the Netherlands would take at least a year.

We regret that we cannot fulfill all of your requests, but we hope that with the changes we have made clear that this paper answers new relevant research questions and is truly an original analysis of a known dataset.

Sincerely yours,

On behalf of Prof. Teus van Laar and Prof. Joost Heutink,

Iris van der Lijn, MSc, PhD candidate

Royal Dutch Visio & University of Groningen

Department of Clinical and Developmental Neuropsychology

Grote Kruisstraat 2/1

9712 TS Groningen

The Netherlands

+316-36319906

i.van.der.lijn@rug.nl
